# Extending More than One Week the Shelf Life of Fresh-Cut Lettuce Using Vinegar Enriched in Bioactive Compounds Encapsulated in α-Cyclodextrins

**DOI:** 10.3390/foods13193142

**Published:** 2024-10-01

**Authors:** María de los Ángeles Martínez-Sánchez, Ginés Benito Martínez-Hernández, Antonio López-Gómez

**Affiliations:** Food Safety and Refrigeration Engineering Group, Department of Agricultural Engineering, Universidad Politécnica de Cartagena, Paseo Alfon4so XIII, 48, 30203 Cartagena, Spain; mariadelosangeles.martinez@edu.upct.es (M.d.l.Á.M.-S.); ginesbenito.martinez@upct.es (G.B.M.-H.)

**Keywords:** encapsulated vinegar powder, polyphenols, antioxidant activity, antimicrobial activity, food safety

## Abstract

Fresh-cut salads are highly appreciated by consumers due to their healthy and convenient nature. Fresh-cut (FC) lettuce is one of the best-selling FC products due to its freshness and low preparation time for salads. However, FC lettuce is very perishable and, in addition, has been recently associated with severe foodborne illness alerts. Alternative natural sanitizing treatments to conventional sodium hypochlorite are needed. The antimicrobial activity of vinegar has been well-known since ancient times. In addition, bioactive compounds from plant byproducts are widely recognized for their antioxidant properties. This work aimed to evaluate the use of a novel and encapsulated vinegar powder enriched with bioactive compounds from fruit byproducts with high antimicrobial and antioxidant properties to preserve the physicochemical (titratable acidity, total soluble solids, weight loss, and color), microbial (psychrophiles, enterobacteria, lactic-acid bacteria, molds, and yeasts), and sensory quality of FC lettuce at 4 °C for up to 10 days. Small to no differences were observed in terms of physicochemical quality (≈0.1% titratable acidity; 2.3–3.3% total soluble solids; <1% weight loss) in comparison to control samples through storage. Vinegar treatments reduced by 2–4 log CFU g^−1^ the microbial loads after 10 days. In addition, a synergistic sensory antibrowning effect (greener and less yellowish appearance, showing −a* and −b*) between organic acids and the released polyphenols from the encapsulated plant byproduct extracts was observed. Hence, the enriched encapsulated vinegar represents an effective green alternative to conventional sanitizers to maintain the quality of FC lettuce through storage time.

## 1. Introduction

Fresh-cut (FC) fruits and vegetables are usually raw products that have been minimally processed with mild operations like cutting, sanitizing treatments, and modified atmosphere packaging, combined with low-temperature storage, to preserve their fresh quality attributes and food safety [1]. The processing of FC products shall not involve any intense thermal processing or freezing. FC products are supplied as fresh nourishment ready-to-eat or ready-to-cook, moreover, defined as ready-to-use due to their raw nature consumption [2,3].

Consumers highly appreciate FC products due to their low preparation time, healthiness (due to their high contents of nutritional and bioactive compounds), and fresh-like characteristics [4,5]. Although the FC consumption in Spain decreased approximately by 25% in 2022 [6], the consumption of FC vegetables increased by 4.9% from 2022 to 2023, with an increment of the market value of 6.9% [7]. Among FC products, leafy vegetables such as FC lettuce (*Lactuca sativa* sp.), for example, are considered the preferred option by many consumers [8,9]. In fact, last year the market value of FC lettuce increased by 2.5%, being the second most consumed vegetable (15.9%) in Spain, only after potatoes (42.4%) [7]. Among lettuce varieties, Iceberg (*L. sativa* var. *capitata*) is the most preferred variety for FC lettuce processing, mostly due to its morphological characteristics and resistance to mechanical damage, agronomical characteristics, all-year availability, pleasant flavor, and crispy texture [10,11,12]. In addition, Iceberg lettuce is mainly composed of water (>95%) while also having high contents of fiber, minerals, phenolic compounds (quercetin, flavonoids, or phenolic acids, among others), and other antioxidant compounds such as ascorbic acid [3,12,13].

Microbial growth during storage of FC products usually does not imply a negative impact on human health, but the incidence of pathogens like *Escherichia coli*, *Salmonella* spp., or *Listeria monocytogenes* may occur, compromising the food safety of these FC products [5,11,14]. Hence, ahead of the distribution and sale of these raw products, effective sanitizing treatments and other complimentary processing operations (modified atmosphere packaging, refrigerated storage, etc.) are needed to control microbial growth. Regarding sensory quality, microbial growth leads to an off-flavor incidence, which is especially associated with the growth of some gram-negative and other spoilage bacteria such as lactic acid bacteria [2,14]. Different factors are involved in contamination pathways in FC vegetables. Some of them involve preharvest adverse conditions via soil or irrigation water contamination, for example, but also postharvest unsuitable human handling or infected processing or packaging equipment [2,11]. Contaminated FC products have been correlated to severe food outbreaks [5,11]. Thus, disinfection treatments of raw leafy products are of major importance to ensure not only their adequate appearance but, above all, their food safety [5]. Minimal processing of FC leafy products usually includes preliminary shredding, followed by pre-washing to remove crop soil and dirt before the main disinfection step before surface drying and packaging [2,3].

Nevertheless, Iceberg lettuce and other similar leafy products are known to be highly perishable products that face sensory and microbial spoilage when cut and stored, causing ineluctably quality reduction [10]. Respiration and transpiration rates are entailed in physical deterioration concerning the metabolic activity of FC lettuce’s tissues and eventually biochemical changes leading to product wilting [13]. Typical color, texture, aroma, and flavors of lettuce are affected as a result of FC processing, which affects product quality and consumer acceptance [2,13]. Marketability and distribution of FC products are thereby affected [15,16]. Certain recurrent issues responsible for FC lettuce senescent deterioration are leaf discoloration and browning associated with chlorophyll loss, enzymatic polyphenol degradation, etc. [17,18]. Cutting during minimal processing also causes tissue damage since the disrupted tissue areas have higher respiration rates due to the abiotic stress generated by cutting. Additionally, the disrupted plant cells release phenolic compounds and other substances that, in addition to being the substrates for the polyphenol oxidase activity (responsible for lettuce browning), are accountable for an unpleasant aroma. In addition, cut tissues are entranceways for microorganisms, which grow and develop on the lettuce tissue, accelerating its deterioration [3,12].

For that reason, FC processing technologies should ensure the extension of the product quality through microbial control while maintaining intrinsic organoleptic-like crispness or firmness, together with its nutritional profile [15]. Different processing strategies have been developed alone or in combination to achieve optimal quality during storage while maintaining sensory and microbial specifications. For instance, modified atmosphere packaging, sanitizing washing with chlorinated water, ozone treatments, ultraviolet light, natural antimicrobial treatments, active packaging, and intelligent packaging are some of the currently available options that are being considered [4,14,15,16]. However, these technologies also carry some limitations. Sodium hypochlorite (NaOCl) and other chlorinated sanitizers are nowadays still widely used in disinfection treatments of minimally processed vegetables [19]. These sanitizers are capable of reducing microbial growth but also encounter important disadvantages concerning the generation of chemical byproducts of a carcinogenic nature [5,11]. On the other hand, although physical methods such as modified atmosphere packaging are quite effective when in combination with cold storage, they may moreover negatively affect the sensory characteristics of FC lettuce [2,8,18]. Moreover, high levels of CO_2_ (during modified atmosphere packaging generation) may even provoke pathogenic bacteria growth [14]. Hence, new alternative sanitizing treatments for NaOCl are needed.

Natural antimicrobial treatments have been proposed in FC vegetables, such as bioactive compounds from plant extracts like organic acids and phenolic compounds, among others [18]. Bioactive compounds from many fruits and vegetables have high antimicrobial and antioxidant properties, for example, anthocyanins from pomegranates, carotenoids from carrots, etc. [18,20,21]. Plant extracts have been effectively used against *Salmonella* spp., *Listeria monocytogenes*, and *Escherichia coli* O157:H7 [22]. The antimicrobial activity of organic acids like lactic, citric, acetic, or tartaric acids on FC products has also been widely reported against coliforms and foodborne pathogens like *Salmonella* spp. [5,14,23]. On this basis, vinegar has been employed as a natural preservative, partly due to its acetic acid content [24]. Hence, sanitizing washing treatments of several fresh fruit and vegetables (lettuce, apple, pineapple, watermelon, cabbage, carrot, cucumber, green pepper, runner beans, and spring onions) with vinegar solutions (containing acetic acid concentrations from 0.5 to 2.5%) showed a 15–82% inhibitory effect on microbial growth, directly proportional to increasing the acetic acid concentration and immersion time (0–10 min) [25]. In particular, Nascimento et al. found that the application of 25–50% vinegar reduced ≈3 log CFU g^−1^ of microbial loads (mesophiles, molds, and yeasts) in fresh lettuce [26]. Other researchers also reported microbial (mesophilic and coliform) reductions in fresh lettuce up to 4 log CFU g^−1^ after washing treatments including vinegar (25%) for 30 min [27].

However, the highly acidic nature of vinegar could potentially affect the organoleptic taste of fresh vegetables with vinegar-related off-flavors [25,28]. In that sense, encapsulation technologies could serve as a satisfactory technique to apply natural bioactive compounds while supplying adequate conditions during FC sanitation [18].

This work aimed to study the application of an innovative encapsulated vinegar enriched with bioactive compounds from fruit byproducts to preserve the physicochemical, microbial, and sensory quality of FC Iceberg lettuce during cold storage at 4 °C. For this purpose, in this investigation two application options were evaluated as sanitizing treatments: direct powder application and dissolved in a washing shower. Vinegar powder was selected as a sanitizing treatment since its transportation and storage imply fewer costs and space needs, together with higher stability compared with stored liquid sanitizing solutions. In order to improve a more homogeneous application of the vinegar powder over the lettuce surface, a washing treatment including the vinegar powder was also studied. In addition, mixing the vinegar powder with water would imply a rapid dissolution in the water due to the hydrophilic nature of the external side of the cyclodextrin molecule. Hence, a release of the encapsulated active compounds is expected due to the dissociation of the vinegar–cyclodextrin inclusion complex due to water molecules [29]. The relatively rapid release of the encapsulated active compounds is desired in FC products due to their very short storage period, while a more controlled release of the encapsulated active compounds is desired in other food products with longer storage times.

## 2. Materials and Methods

### 2.1. Materials and Treatment Preparation

Lettuce (*Lactuca sativa* L. var. *capitata* “Iceberg”) heads were farmed by Agrupapulpí S.A. (Almería, Spain). Freshly harvested samples, free of visual damages, were selected and transported to the pilot plant of the Agricultural Engineering Department of the Universidad Politécnica de Cartagena, where they were immediately processed as described below. Liquid distilled vinegar was provided by the company JR Sabater S.A. (Murcia, Spain). Fruit byproduct extract powder (rich in naturally occurring bioactive compounds in those fruits, mainly polyphenols) was provided by Agrosingularity S.A. (Murcia, Spain). α-cyclodextrin was obtained from Wacker Chemie AG (Burghausen, Germany).

Vinegar encapsulation was made by Bioencapsulation and iPackaging S.L. (Fuente Álamo, Spain) to obtain the non-buffered or buffered enriched vinegar powder (Table 1). For it, before spray drying, byproduct extract powder (2%) was dissolved in liquid vinegar (either non-buffered or buffered), and then α-cyclodextrin at 10% (*w*/*w*) was added and dissolved. α-cyclodextrin was used as a coating material for the spray-drying process to obtain the encapsulated vinegar.

**Figure 1:**
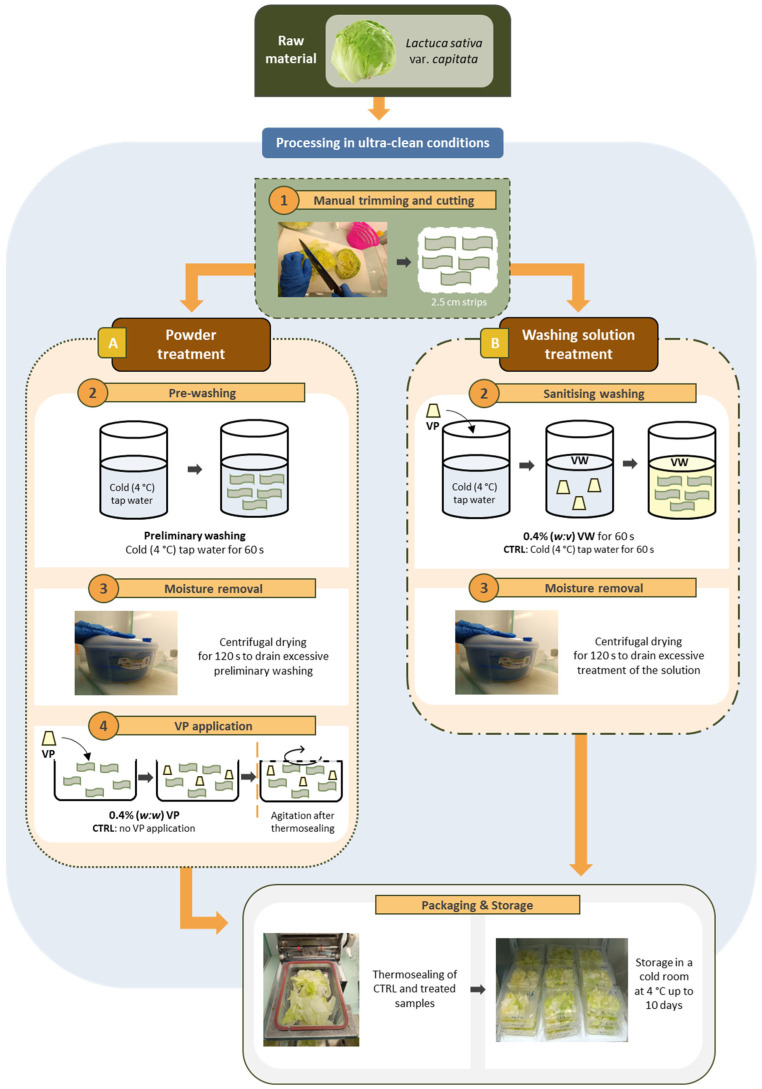
Flowchart of FC lettuce processing under ultraclean conditions (8 °C), using the proposed sanitizing treatments: enriched vinegar in powder form (**A**) or as a washing solution (**B**). VP = vinegar powder; VW = vinegar washing; CTRL = control treatment. Arrows indicate the addition of VP.

The studied treatments were direct in-package application of the encapsulated vinegar powder (Non-buffered Vinegar Powder (N-VP) or Buffered Vinegar Powder (B-VP)) on lettuce samples (Figure 1A; Table 1); and sanitizing washing shower of lettuce samples with the encapsulated vinegar powder (Non-buffered Vinegar Washing (N-VW) or Buffered Vinegar Washing (B-VW)) (Figure 1B; Table 1). For the (A) treatment application (as described with more detail in Section 2.2), encapsulated vinegar was directly applied to the lettuce sample at a 2:50 ratio (vinegar powder weight (g)–lettuce weight (g)). For the (B) treatment application, lettuce was washed in water containing 0.4% of the vinegar powder at a 1:20 ratio (lettuce weight (g)–washing treatment volume lettuce (mL)). Powder and sanitizing washing doses were adjusted based on preliminary analyses to optimize the highest doses that did not affect the sensory quality of the product.

### 2.2. Processing and Storage Conditions

Lettuce processing (≈4 kg) and packaging were conducted at 8 °C in ultra-clean conditions inside a laminar flow cabinet (ISO 5; equivalent to 100 FED STD 209E class) located in a clean room (ISO 7; equivalent to 10,000 FED STD 209E class) to reduce microbial contamination risk after sanitation treatment [30]. Firstly, external leaves from the lettuce head were hand-removed, and then fresh samples were manually trimmed and later shredded into 2.5 cm long uniform strips using a domestic stainless-steel knife. Lettuce strips were mixed to be homogenous and representative and afterward separated into five different ≈ 750 g lots that were subjected to two different sanitation procedures (Figure 1A,B). For sanitizing powder treatments (A), lettuce strips were immersed in cold tap water (4 °C) (250 g of lettuce in 1 L water) for 60 s. Excess water was removed from samples using a manual salad centrifuge before packaging.

For the application of vinegar powder treatments (Figure 1A), 0.2 g of VP was placed along with 50 g of drained lettuce strips (to achieve a 2:50 ratio of vinegar powder weight (g)–lettuce weight (g)) in a transparent polypropylene rectangular tray (173 × 120 × 38 mm; 0.75 L capacity) and thermally sealed on the top with a film (EOP616B film, Cryovac^®^; Fuenlabrada, Spain; thickness 39 µm; permeabilities: O_2_, 7.0 cm^3^ m^−2^ day^−1^ atm^−1^; CO_2_, 25.0 cm^3^ m^−2^ day^−1^ atm^−1^; N_2_, 0.5 cm^3^ m^−2^ day^−1^ atm^−1^; water, 10.0 g m^−2^ day^−1^) using a manual tray sealer (La Barket BP 40/E, befor; Sain Priest, France).

For sanitizing washing treatments (Figure 1B), lettuce strips were submerged for 60 s in water with 0.4% of encapsulated vinegar to achieve a 1:20 ratio of lettuce weight (g)–washing treatment volume lettuce (mL). Excess water removal and packaging were made as previously described. Subsequently, 50 g of treated samples were packaged in the plastic trays as previously described.

Lettuce strips washed with cold tap water, with no addition of encapsulated vinegar, were used as the control treatment (CTRL). Unwashed control was not considered in this study since common sanitizing treatments of lettuce already include a washing step [31]. Three biological replicates (i.e., three trays) per treatment and sampling time were prepared.

Finally, samples were stored in a cold room at 4 °C under dark conditions for up to 10 days. At every sampling time (0, 1, 3, 7, and 10 days), samples were analyzed for weight loss, pH, titratable acidity, total soluble solids, color, microbial quality, and sensory quality.

### 2.3. pH, Titratable Acidity, Total Soluble Solids, and Weight Loss

The soluble solid content, pH, and titratable acidity of samples were determined on a prepared juice made with distilled water (lettuce–water in 1:10, *w–volume*) with a household blender (ErgoMixx 600 W, BSH Electrodomésticos; Zaragoza, Spain), followed by 4-layer cheesecloth. The pH of the obtained juice was measured with a digital pH meter (GLP 21, Crison; Barcelona, Spain). Titratable acidity was determined by titration until pH 8.1 with 0.1 M NaOH using an automated titrator (916 Ti-Touch, Metrohm; Herisau, Switzerland), and results were expressed in % (g milliequivalents of malic acid per 100 mL). Total soluble solids content was measured using a digital refractometer (Atago model PAL-1; Tokyo, Japan).

The weight loss of samples was determined by weighing all trays containing the samples at each sampling time. The weight loss of lemons was calculated as follows (Equation (1)):

(1) $$Weight\;loss\;\left(\%\right)=\frac{\left(W_{0}-W_{t}\right)}{W_{0}}\times 100$$

where *w*_0_ is the initial weight sample (at day 0) and *w_t_* is the sample weight at each sampling time.

### 2.4. Color Measurement and Browning Determination

The surface color of lettuce strips was measured using a colorimeter (CR-400 Chroma Meter, Konica Minolta; Tokio, Japan) at CIE illuminant C and 2° observer with an 8-mm diameter measuring aperture. The colorimeter was previously calibrated using a calibration white plate (Y = 94.3; x = 0.3142; y = 0.3211). Color values (L*, a*, and b*) of each sample were obtained as the mean value of three superficial readings per replicate. Color changes and browning occurrence through storage were calculated as the Total Color Differences (ΔE*) using Equation (2) [29].

(2) $$\Delta E^{*}=\sqrt{{\left(\Delta L^{*}\right)}^{2}+{\left(\Delta a^{*}\right)}^{2}+{\left(\Delta b^{*}\right)}^{2}}$$

### 2.5. Microbiological Analyses

The microbiological quality of samples was assessed by total psychrophiles, enterobacteria, lactic acid bacteria, molds, and yeast counts determination. For microbial analysis, 25 g of lettuce strips were mixed with 225 mL of buffered peptone water (pH 7; Scharlau Chemie SA; Barcelona, Spain) inside a *stomacher* bag (model 400 Bags 6141; London, UK) [26]. Subsequently, 10-fold dilution series were made with buffered peptone water. Then, aliquots of 1 mL were pour-plated into Plate Count Agar (PCA) (Scharlau Chemie SA; Barcelona, Spain) for microbial count of psychrophiles (4 °C/7 days), Violet Red Bile Dextrose Agar (VRBD) (Scharlau Chemie SA; Barcelona, Spain) for enterobacteria (37 °C/48 h), and De Man Rogosa and Sharpe (MRS) (Scharlau Chemie SA; Barcelona, Spain) for lactic-acid bacteria (30 °C/48 h). For molds and yeasts, aliquots of 0.1 mL were spread-plated onto Rose Bengal Agar (Scharlau Chemie SA; Barcelona, Spain) (25 °C/7 days). Results were reported as log of colony-forming units (CFU) per gram (log CFU g^−1^).

### 2.6. Sensory Quality Determination

The sensory analyses were made by a panel of 7 untrained members (60% women, 40% men; aged 20–50 years) consisting of researchers and students from the Plant Biotechnology Institute (Universidad Politécnica de Cartagena). Each panelist was instructed to examine the appearance, aroma, flavor, texture, and general acceptance using a 5-point hedonic scale of acceptability (5: excellent; 3: acceptable; 1: extremely bad) [31], based on the scoring of lettuce descriptors from [5], with some modifications. Sensory evaluation was conducted in a standardized sensory room [32] equipped with individual tasting booths to determine quality evolution over refrigerated storage. For it, samples were placed (≈5 g) in aluminum containers coded with random 3-digit labels. Mineral still water and unsalted breadsticks were also provided to panelists to clean the palate between samples.

### 2.7. Statistical Analysis

Results were statistically processed using RStudio software (v.4.2.3 Posit Software; Boston, MA, USA). Statistical analysis was performed at a 95% confidence level (*p* < 0.05) for unidirectional ANOVA and Tukey HSD test SPSS Software (v.19 IBM; New York, NY, USA). Data are expressed as the mean value of three replicates with its standard deviation.

## 3. Results and Discussion

### 3.1. pH, Titratable Acidity, Total Soluble Solids, and Weight Loss

The titratable acidity (TA) and total soluble solids (TSS) of FC lettuce are shown in Table 2. During the processing day, the pH of all samples was close to neutral values (5.7–6.4; Supplementary material Table S1), similar to the results reported by other authors for fresh processed lettuce [9]. The use of unbuffered normal (acidic) VP (N-VP) induced the lowest pH value, probably because of the partial release of the encapsulated acetic acid upon contact with the lettuce surface, due to dissociation of the inclusion complex at high relative humidity conditions [33]. Contrarily, the VP treatments in solution (N-VW and B-VW) presented no differences (*p* > 0.05) with the CTRL treatment. The observed pH values are in accordance with the literature, which is linked with a typical low-acidity nature in lettuce [34]. Hence, the TA of samples was approximately 0.1% (Table 2) with no differences (*p* > 0.05) between treatments and adequately correlated to the neutral pH observed.

In addition, TA (Table 2) did not show differences (*p* > 0.05) after 3 days of storage and barely showed slight differences between treatments through refrigerated storage, as previously observed [12]. The neutral acidity of lettuce, together with the high proportion of water in its tissues, favors its microbiological deterioration [3]. This buffering effect for the product acidity of lettuce after the application of vinegar treatments could favor a decrease in microbial growth (see microbial data), favoring better preservation (see sensory data) during storage [35,36].

Sanitation treatments did not affect the total soluble solids content, ranging values from 2.3–3.0% at day 0 (Table 2), being consistent with other authors (3.8–4.0%) [37]. Firstly, there was observed a decrease (*p* > 0.05) in TSS after 3 days of storage in CTRL samples, probably due to an increase in microbial growth rate [37]; however, we monitored a rise (*p* < 0.05) in TSS of CTRL samples as storage time increased, this accumulation being often related to lettuce deterioration [18]. After 7 days of refrigerated storage, TSS content was not different among treatments (*p* > 0.05), although differences were noticed at day 10 of storage when a significant depletion occurred in TSS of buffered treatments (B-VP and B-VW), positively correlated with an increase in microbial loads (Table 3).

**Figure 2:**
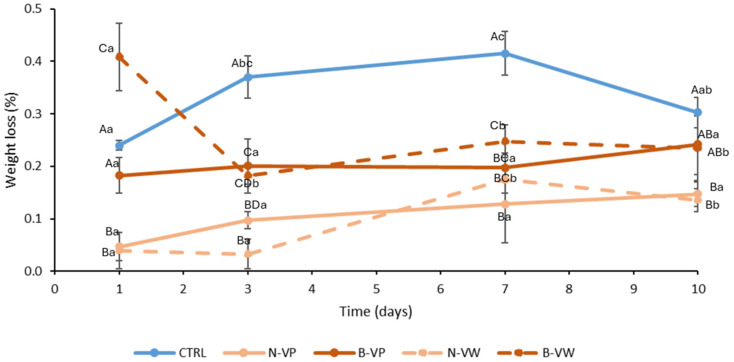
Weight loss of control (CTRL) and treated fresh-cut lettuce during cold storage at 4 °C for up to 10 days (mean (n = 3) ± standard deviation). CTRL, control (absence of encapsulated vinegar); N-VP, normal (acidic) enriched vinegar powder; B-VP, buffered enriched vinegar powder; N-VW, normal (acidic) enriched vinegar powder washing solution; B-VW, buffered enriched vinegar powder washing solution. Different uppercase letters denote significant differences (*p* < 0.05) among different treatments for the same storage time. Different lowercase letters denote significant differences (*p* < 0.05) among different storage times for the same treatment.

The weight loss of samples was very low (<1%), as expected in a packaged FC product (Figure 2). The weight loss results we report here were noticeably lower than those investigated for refrigerated FC stem lettuce (0.8–3.5%) for 0–6 days and pulsed-light treated FC lettuce (1.4–13.2%) for 0–8 days [37,38]. In particular, treatments showed lower weight losses (<0.25%) compared with CTRL (0.25–0.4%) during storage. Among them, VP treatments (N-VP and B-VP) showed more stable values during storage since no differences (*p* > 0.05) were observed during storage for these treatments, so the preservation conditions were considered adequate since an absence of weight loss is related to better quality maintenance [34]. Interestingly, an initial marked weight loss from days 1 to 3 was observed for the B-VW treatment, which may be explained by an osmotic phenomenon as a consequence of washing with this particular treatment, including buffered vinegar (acetates released after solubilizing the encapsulated vinegar), leading to such weight loss. Weight losses tended to intensify from day 3 to day 7 for vinegar solution treatments (N-VW and B-VW), although reached values at day 10 were similar to powder treatments. This increase in weight loss is usually attributable to the normal metabolic wilting of refrigerated lettuce through storage [34]. Hence, low weight loss of samples may be linked to well-preserved tissue turgor, which has been widely reported as an important sensory attribute in lettuce tissues [13]. Since a high transpiration rate is associated with the deterioration of lettuce, vinegar sanitation could have shown a lowering effect on the respiration of tissues, thus resulting in lower weight loss.

### 3.2. Color

The Total Color Differences (ΔE*) values are shown in Table 2. Browning was observed throughout storage with decreasing L* values for the powdered treatments (N-VP and B-VP), which was also correlated with increased ΔE*. However, this trend was not observed in N-VW and B-VW treatments, probably due to the removal effect of phenolic compounds achieved by the washing procedure, whose accumulation after cutting during processing is known to be responsible for browning in FC lettuce [18].

Color differences (ΔE*) were lowered (*p* < 0.05) in N-VW treatment during storage since polyphenols from enriched vinegar washing treatment inhibited browning pigment formation, achieving better protection of the sample color, with less browning being positively correlated to better quality maintenance during storage (See Section 3.4), as stated by other researchers [10,22]. The observed decrease (*p* < 0.05) in color changes of CTRL treatment during storage agrees with previous studies that showed that control washing with water did not induce color differences in FC lettuce as L* values were not altered [5]. However, powder treatments increased the surface color changes at the end of storage time, mainly due to enzymatic browning, as stated by other researchers that related browning incidence with color changes in leaves [10,29]. The decrease in color changes of lettuce washed with 0.4% enriched vinegar throughout refrigerated storage reflected adequate preservation and proper maintenance of lettuce freshness, in accordance with results reported by other authors [29].

Browning has also been correlated in lettuce with tissue damage since it triggers the enzymatic activity of phenylalanine ammonia-lyase (PAL). In particular, the enzymatic activity of PAL leads to the synthesis of different hydroxycinnamic acids and triggers a complex pathway that eventually involves the hydroxylation of monophenols to *o*-diphenols and their subsequent oxidation to *o*-quinones by polyphenol oxidase (PPO) [4,17,18]. Contrary to powder treatments, washing treatments did not induce high L* changes during storage, which led to lower L* values in these samples after 10 days. Among treatments, N-VW and B-VW showed the lowest color variations with ΔE* values of 6.7 ± 3.6 and 8.6 ± 2.0, respectively, after 10 days. These results reflected less browning and adequate preservation of refrigerated lettuce strips owing to the washing effect of compounds related to enzymatic browning as well as an antioxidant effect of bioactive compounds from enriched vinegar that may have inhibited enzymatic activity itself [9,18]. In fact, acetic acid and its salts have been demonstrated to have an inhibiting effect on PAL, thus reducing the phenolic content and browning incidence of lettuce [39].

Control lettuce showed a reddish hue (+a*) (*p* < 0.05) (Supplementary material Table S1) with increasing storage time due to the appearance of an oxidized appearance and a deterioration of the visual quality of plant tissues related to a loss of chlorophyll [4,9,13,29]. Samples treated by immersion in the enriched vinegar aqueous solution maintained the greenish hue (−a*) of lettuce and a better appearance for 10 days under refrigeration. However, no differences (*p* > 0.05) were found with respect to the rest of the treatments. The antioxidant activity of the bioactive compounds released from the encapsulated vinegar probably controls sample browning, contributing to an increase in the quality of samples during cold storage [3,40]. Washing with unbuffered enriched vinegar provided a slightly acidic environment due to the presence of acetic acid, which, in combination with the antioxidant effect of polyphenols, limited the enzyme activity responsible for browning [40]. The differences found in the yellowing (+b*) (Supplementary material Table S1) of lettuce on the initial day could be due to the heterogeneity of the samples. In general, the unbuffered treatments showed higher b* values than the rest of the samples. It indicated that the buffered vinegar treatment, similar to CTRL samples, was able to preserve the color of samples without the appearance of browning/yellowing [3].

### 3.3. Microbiological Quality

The microbiological results of total psychrophilic, enterobacteria, and lactic acid bacteria are shown in Table 3. Psychrophile counts showed significant differences as a function of vinegar type (unbuffered or buffered) and the application type (Table 3). At day 0, total psychrophilic counts ranged from 1.0 to 3.6 log CFU g^−1^. The application of enriched vinegar encapsulated in solution showed significant differences (*p* < 0.05) with respect to the direct use of VP and CTRL. Such immersion in the solution studied achieved an immediate antimicrobial effect, observing a reduction ≈ 1.5 log CFU g^−1^ with respect to CTRL, which may be associated with the polyphenol released after the dissolution of the enriched vinegar [22]. The use of unbuffered or buffered vinegar did affect the total psychrophilic counts, regardless of the treatment applied. The addition of unbuffered VP showed no difference (*p* > 0.05) with CTRL, since washing with water has been reported to be effective in reducing (1.4–2.5 log CFU g^−1^) microbial counts on the surface of fresh products [41].

At day 3, total psychrophile counts increased (*p* < 0.05) in CTRL samples, reaching 5.6 log CFU g^−1^. The acidic nature of the unbuffered fortified vinegar slowed psychrophilic growth, especially in the case of the N-VW treatment (1.0 ± 0.1 log CFU g^−1^). The antimicrobial activity of acetic acid has been widely reported by other authors [24,27,28]. A progressive increase in psychrophile counts was observed during refrigerated storage. However, washing with the enriched vinegar solution maintained counts at levels of ≈3 log CFU g^−1^ after 7 days. The N-VW treatment maintained total psychrophilic counts (1.0 ± 0.1 log CFU g^−1^) during 3 days of storage, with counts of ≈3.1 log CFU g^−1^ after 7 days. The rest of the treatments, in contrast, approached and exceeded the consumption limit generally considered safe (>7 log CFU g^−1^) [4]. These results demonstrated the strong antimicrobial potential of the fortified vinegar under study when applied in solution, maintaining the product’s microbial quality beyond 10 days (3.0–4.8 log CFU g^−1^).

Washing with the enriched vinegar solution achieved a significant ≈ 2 log CFU g^−1^ reduction for enterobacterial counts (Table 3) at day 0. This reduction was similar to that achieved after the use of a 25% vinegar solution [27]. During storage, an increase (*p* < 0.05) in enterobacterial counts was observed in CTRL and powder treatments with ≈4 log CFU g^−1^ at day 1, exceeding the 7 log CFU g^−1^ after 7 days at 4 °C. This increase is in agreement with results reported by other researchers, who stated that lettuce spoilage is due to the proliferation of gram-negative bacteria [4,42]. On the other hand, washing in the unbuffered or buffered enriched vinegar solutions controlled the enterobacteria loads (1.0–3.0 log CFU g^−1^) during the first three days of storage. The unbuffered solution barely reached 2.8 log CFU g^−1^ after 10 days, demonstrating adequate preservation of the FC lettuce.

Lactic acid bacteria counts were found to be below the detection limit (<1.0 log CFU g^−1^) throughout storage in treatments with the enriched vinegar solution (Table 3). These results were consistent with other authors who found that the growth of these microorganisms was significantly lower than that of gram-negative bacteria due to the nutritional composition of lettuce itself [42]. However, the significant increase in lactic acid bacteria counts at the end of storage in powdered treatments was related to a loss of the FC lettuce quality, probably as a result of the proliferation of bacteria of the genus *Leuconostoc* [43,44]. The progressive release of the acetic acid encapsulated in the VP could have contributed, together with the organic acids produced by lactic acid bacteria, to the deterioration of the FC lettuce. In contrast, immersion in the enriched vinegar solution resulted in a homogeneous distribution of the whole composition on the plant tissue, with the acetic residues evaporating during the drying step of the lettuce before packaging, avoiding damage to the lettuce.

On the other hand, the counts of molds and yeasts (Supplementary material Table S2) were found to be below the detection limit (<2.0 log CFU g^−1^) during the entire storage. It may be explained due to the low growth rate of these microorganisms in vegetables, such as lettuce, compared with other microorganisms responsible for product deterioration [4,42].

### 3.4. Sensory Quality

**Figure 3:**
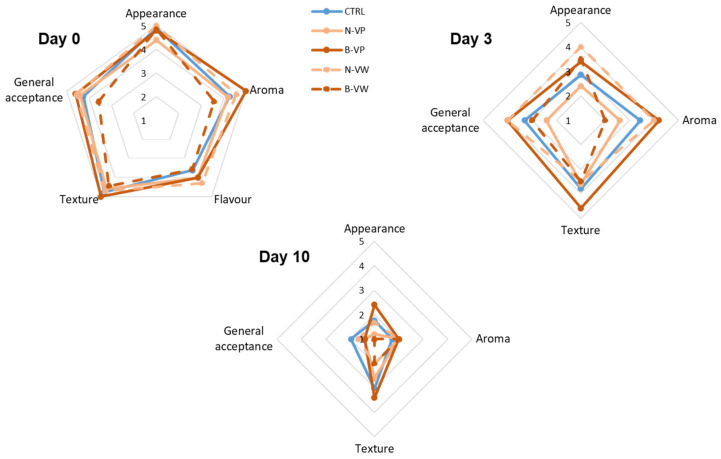
Sensory evaluation of control (CTRL) and treated fresh-cut lettuce during cold storage at 4 °C for up to 10 days (mean (n = 3) ± standard deviation). CTRL, control (absence of encapsulated vinegar); N-VP, normal (acidic) enriched vinegar powder; B-VP, buffered enriched vinegar powder; N-VW, normal (acidic) enriched vinegar powder washing solution; B-VW, buffered enriched vinegar powder washing solution.

**Figure 4:**
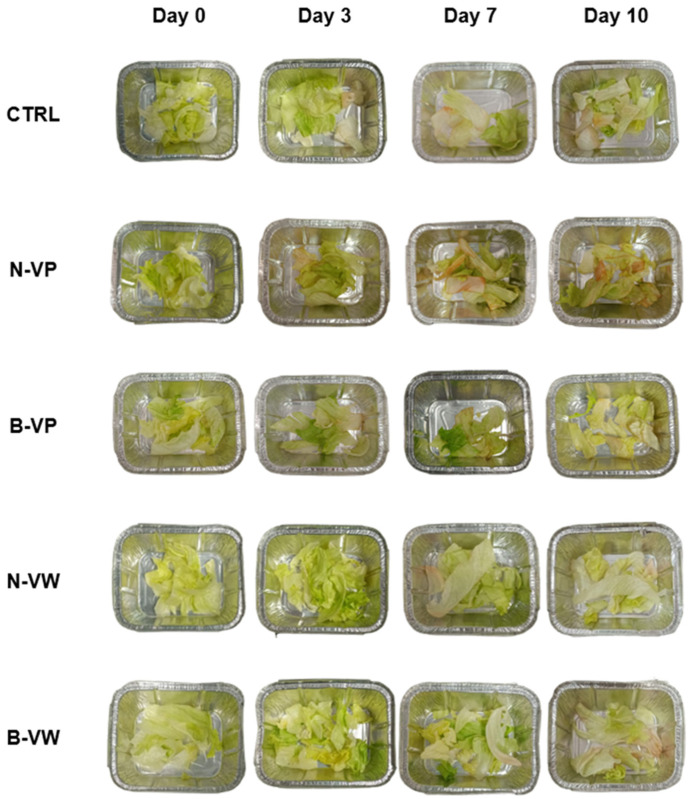
Fresh-cut lettuce evolution during cold storage at 4 °C for up to 10 days. CTRL, control (absence of encapsulated vinegar); N-VP, normal (acidic) enriched vinegar powder; B-VP, buffered enriched vinegar powder; N-VW, normal (acidic) enriched vinegar powder washing solution; B-VW, buffered enriched vinegar powder washing solution.

The evolution of the sensory quality of FC lettuce during days 0, 3, and 10 of storage can be observed in Figure 3 and Figure 4. At day 0, the lettuce appearance was adequate, reaching high scores of 4.4–5.0 points. No differences (*p* > 0.05) were found between CTRL or the application of VP or VW. It may indicate that processing and vinegar treatment did not affect the visual quality of FC lettuce, an important parameter to consider in minimally processed fresh produce [45]. The aroma and flavor of CTRL were neutral and insipid, while the enriched vinegar treatment highlighted its freshness, contributing to its overall acceptability (4.4–4.6 points). The enriched vinegar treatment did not communicate sour notes to the lettuce or any off-flavors or aromas. However, the buffered enriched vinegar wash did result in slightly more astringency and bitterness, although it did not detract from its acceptability to the panelists who found the treatment acceptable (3.57 points). The texture of lettuce was not affected and showed no difference between CTRL or the application of enriched vinegar in powder form or by washing solution.

As the storage time progressed (day 3), a deterioration in the sensory quality of samples was observed, especially in terms of appearance (Figure 4). The N-VP and buffered vinegar treatments also showed a deterioration in their visual quality, which is usual in FC vegetables [9]. On the other hand, the N-VW treatment maintained a considerably good appearance (4.0 points), controlling the appearance of oxidations leading to the absence of browning and high acceptance of samples. Despite the visual detriment during storage, samples were still considered acceptable (3.0–4.0 points), except for the N-VP treatment (2.4 points). It could be due to the release of the encapsulated acetic acid together with the bioactive compounds, which led to the appearance of reddish and more yellowish shades (+a* and +b*) concerning the processing day; this visual deterioration impaired their acceptability [13].

At the end of storage time (day 10), evidence of yellow areas and discoloration of the tissues along with obvious oxidized areas drastically affected the sensory perception of the FC lettuce, since lower scores were obtained for these two attributes (appearance and general acceptance) in comparison to processing day, like has also been reported by other authors [9]. After 10 days, lettuce appearance was severely impaired (1.0–2.4 points). The appearance of the N-VW treatment showed no difference (*p* > 0.05) with respect to CTRL, although it reduced texture, which decreased its overall acceptability [14]. Washing with N-VW slowed lettuce deterioration, although it failed to prevent sensory loss as evidenced by other researchers during the storage of washed FC vegetables [9]. The aromatic profile of lettuce treated with enriched vinegar decreased, although it was slightly more adequate than CTRL, which presented unpleasant aromas related to enzymatic damage and oxidations typical of senescence-associated processes in FC lettuce [3,14]. The N-VW treatment showed the best appearance and general acceptability with respect to the rest of the vinegar-enriched treatments after 10 days of storage (Figure 4).

## 4. Conclusions

Processing and production of FC vegetables like Iceberg lettuce represent a challenge for the FC industry in terms of maintaining freshness and visual quality while ensuring food safety as well. In this experimental study, the antioxidant and antimicrobial effects over FC lettuce of a novel encapsulated vinegar enriched with polyphenols from fruit by-products and encapsulated within alpha-cyclodextrins were investigated. According to our results, it has been demonstrated that sanitizing with a vinegar powder washing solution was effective for microbial control during refrigerated storage of FC lettuce. Encapsulated vinegar treatment significantly preserved the microbial quality of samples by reducing total psychrophiles (1.5–3.3 log CFU g^−1^), enterobacteria (1.1–3.5 log CFU g^−1^), lactic acid bacteria, molds, and yeasts during storage (both below the detection limit). It has also been demonstrated its antioxidant potential via synergistic effect along with fruit polyphenol enrichment in browning control (−a* and −b*), leading to lower ΔE* values (6.7 ± 3.6) similar to control (5.2 ± 1.5) after 10 days, resulting in better visual appearance (≈2.0 points) and texture (2.6–3.4 points) by the end of storage time, in comparison to control samples (2.0 and 3.0 points, respectively). Thus, the use of enriched and encapsulated vinegar could significantly extend the cold storage period of FC Iceberg lettuce, owing to the satisfactory maintenance of the physicochemical, microbial, and sensory characteristics of refrigerated samples. As a result, it can be concluded that encapsulated vinegar enriched with bioactive compounds from fruit by-products (like the pomegranate, which is rich in polyphenol compounds) could represent a natural alternative to conventional disinfectants to preserve the quality and safety of FC vegetables during cold storage for more than one week.

## Figures and Tables

**Table 1:** Sample identification according to sanitation procedure with control (cold tap water) or encapsulated enriched vinegar (conventional or buffered) treatment.

Treatment	Application	Nomenclature
❖ Control	washing	CTRL
❖ Encapsulated vinegar		
Non-buffered Vinegar	Powder	N-VP
	Washing	N-VW
Buffered Vinegar	Powder	B-VP
	Washing	B-VW

**Table 2:** Titratable acidity (TA), total soluble solids (TSS), and total color differences (ΔE*) of control (CTRL) and treated fresh-cut lettuce during cold storage at 4 °C for up to 10 days (mean (n = 3) ± standard deviation).

Time (Days)	Treatment *	TA (%)	TSS (%)	ΔE*
0	CTRL	0.1 ± 0.1 ^ABa^	2.6 ± 0.2 ^Aa^	-
	N-VP	0.1 ± 0.1 ^Ba^	2.3 ± 0.6 ^Aa^	-
	B-VP	0.1 ± 0.1 ^ABac^	3.0 ± 0.1 ^Aab^	-
	N-VW	0.1 ± 0.1 ^Aa^	3.0 ± 0.1 ^Aab^	-
	B-VW	0.1 ± 0.1 ^Aab^	2.7 ± 0.6 ^Aa^	-
1	CTRL	0.1 ± 0.1 ^Aa^	2.8 ± 0.2 ^Aab^	7.0 ± 3.5 ^Aa^
	N-VP	0.1 ± 0.1 ^Aa^	2.7 ± 0.6 ^Aa^	4.5 ± 0.9 ^Aa^
	B-VP	0.1 ± 0.1 ^Aa^	3.0 ± 0.1 ^Aab^	9.1 ± 2.5 ^Aab^
	N-VW	0.1 ± 0.1 ^Aa^	2.3 ± 0.6 ^Aa^	10.0 ± 4.5 ^Aa^
	B-VW	0.1 ± 0.1 ^Aa^	3.0 ± 0.1 ^Aa^	9.8 ± 4.8 ^Aa^
3	CTRL	0.1 ± 0.1 ^Aa^	2.2 ± 0.4 ^Aa^	5.1 ± 0.9 ^Aa^
	N-VP	0.1 ± 0.1 ^Aa^	2.3 ± 0.6 ^Aa^	7.9 ± 1.6 ^Aab^
	B-VP	0.1 ± 0.1 ^Ab^	2.3 ± 0.6 ^Aa^	5.0 ± 2.3 ^Aa^
	N-VW	0.1 ± 0.1 ^Aa^	3.0 ± 0.1 ^Aab^	8.7 ± 3.0 ^Aa^
	B-VW	0.1 ± 0.1 ^Aab^	3.0 ± 0.1 ^Aa^	5.8 ± 1.5 ^Aa^
7	CTRL	0.1 ± 0.1 ^ACa^	2.4 ± 0.2 ^Aa^	4.7 ± 2.4 ^Aa^
	N-VP	0.1 ± 0.1 ^ACa^	2.7 ± 0.6 ^Aa^	9.3 ± 1.6 ^Ab^
	B-VP	0.1 ± 0.1 ^Bc^	3.7 ± 0.6 ^Ab^	6.8 ± 1.4 ^Aab^
	N-VW	0.1 ± 0.1 ^Aa^	3.3 ± 0.6 ^Ab^	8.6 ± 2.9 ^Aa^
	B-VW	0.1 ± 0.1 ^BCab^	3.3 ± 0.6 ^Aa^	7.3 ± 3.4 ^Aa^
10	CTRL	0.1 ± 0.1 ^Aa^	3.3 ± 0.1 ^Ab^	5.2 ± 1.5 ^Aa^
	N-VP	0.1 ± 0.1 ^Aa^	3.0 ± 0.1 ^ABa^	10.4 ± 1.9 ^Ab^
	B-VP	0.1 ± 0.1 ^Aa^	2.3 ± 0.6 ^Ba^	10.3 ± 1.1 ^Ab^
	N-VW	0.1 ± 0.1 ^Aa^	3.0 ± 0.1 ^ABab^	6.7 ± 3.6 ^Aa^
	B-VW	0.1 ± 0.1 ^Ab^	2.3 ± 0.6 ^Ba^	8.6 ± 2.0 ^Aa^

* CTRL, control (absence of encapsulated vinegar); N-VP, normal (acidic) enriched vinegar powder; B-VP, buffered enriched vinegar powder; N-VW, normal (acidic) enriched vinegar powder washing solution; B-VW, buffered enriched vinegar powder washing solution. Different uppercase letters denote significant differences (*p* < 0.05) among different treatments for the same storage time. Different lowercase letters denote significant differences (*p* < 0.05) among different storage times for the same treatment.

**Table 3:** Microbial loads (log CFU g^−1^) of total psychrophiles, enterobacteria, and lactic acid bacteria of control (CTRL) and treated fresh-cut lettuce during cold storage at 4 °C for up to 10 days (mean (n = 3) ± standard deviation).

Time (Days)	Treatment *	Psychrophiles	Enterobacteria	Lactic Acid Bacteria
0	CTRL	2.5 ± 0.3 ^Aa^	3.1 ± 0.3 ^ABa^	1.0 ± 0.1 ^Aa^
	N-VP	2.9 ± 0.3 ^Aa^	3.9 ± 0.4 ^Aa^	1.0 ± 0.1 ^Aa^
	B-VP	3.6 ± 0.2 ^Ba^	2.5 ± 0.6 ^BCa^	1.0 ± 0.1 ^Aa^
	N-VW	1.0 ± 0.1 ^Ca^	1.3 ± 0.6 ^Ca^	1.0 ± 0.1 ^Aa^
	B-VW	1.0 ± 0.1 ^Ca^	2.0 ± 0.1 ^BCa^	1.0 ± 0.1 ^Aa^
1	CTRL	2.8 ± 0.2 ^Aa^	4.4 ± 0.4 ^Ab^	1.0 ± 0.1 ^Aa^
	N-VP	2.9 ± 0.1 ^Aa^	3.7 ± 0.5 ^Aa^	1.0 ± 0.1 ^Aa^
	B-VP	4.5 ± 0.2 ^Bb^	4.6 ± 0.1 ^Ab^	1.0 ± 0.1 ^Aa^
	N-VW	1.0 ± 0.1 ^Ca^	1.0 ± 0.1 ^Ba^	1.0 ± 0.1 ^Aa^
	B-VW	1.5 ± 0.2 ^Da^	1.6 ± 0.6 ^Ba^	1.0 ± 0.1 ^Aa^
3	CTRL	5.6 ± 0.1 ^Ab^	5.2 ± 0.3 ^Ac^	1.4 ± 0.1 ^Ab^
	N-VP	4.7 ± 0.2 ^Ab^	5.6 ± 0.8 ^Ab^	1.0 ± 0.1 ^Aa^
	B-VP	5.8 ± 0.6 ^Ac^	5.0 ± 0.2 ^Ab^	1.3 ± 0.6 ^Aa^
	N-VW	1.0 ± 0.1 ^Ba^	1.0 ± 0.1 ^Ba^	1.0 ± 0.1 ^Aa^
	B-VW	2.1 ± 0.8 ^Bab^	3.0 ± 0.2 ^Cb^	1.4 ± 0.8 ^Aa^
7	CTRL	5.9 ± 0.4 ^Ab^	5.9 ± 0.1 ^Acd^	1.4 ± 0.1 ^Ab^
	N-VP	7.5 ± 0.6 ^Bc^	7.4 ± 0.4 ^Bc^	1.0 ± 0.1 ^Aa^
	B-VP	6.7 ± 0.2 ^ABd^	7.2 ± 0.2 ^Bc^	3.5 ± 0.4 ^Bb^
	N-VW	3.1 ± 0.2 ^Cb^	3.2 ± 0.2 ^Cb^	1.0 ± 0.1 ^Aa^
	B-VW	3.0 ± 0.1 ^Cb^	4.0 ± 0.1 ^Dc^	1.0 ± 0.1 ^Aa^
10	CTRL	6.3 ± 0.3 ^Ab^	6.3 ± 0.1 ^Ad^	1.5 ± 0.1 ^Ab^
	N-VP	7.8 ± 0.2 ^Bc^	7.5 ± 0.1 ^Bc^	5.2 ± 0.4 ^Bb^
	B-VP	7.1 ± 0.2 ^ABd^	7.5 ± 0.1 ^Bc^	3.4 ± 0.6 ^Cb^
	N-VW	3.0 ± 0.8 ^Cb^	2.8 ± 0.4 ^Cb^	1.0 ± 0.1 ^Aa^
	B-VW	4.8 ± 0.7 ^Dc^	5.2 ± 0.2 ^Dd^	1.0 ± 0.1 ^Aa^

* CTRL, control (absence of encapsulated vinegar); N-VP, normal (acidic) enriched vinegar powder; B-VP, buffered enriched vinegar powder; N-VW, normal (acidic) enriched vinegar powder washing solution; B-VW, buffered enriched vinegar powder washing solution. Different uppercase letters denote significant differences (*p* < 0.05) among different treatments for the same storage time. Different lowercase letters denote significant differences (*p* < 0.05) among different storage times for the same treatment.

## Data Availability

The original contributions presented in the study are included in the article/Supplementary Material. Further inquiries can be directed to the corresponding author.

## Supplementary Materials

The following supporting information can be downloaded at: https://www.mdpi.com/article/10.3390/foods13193142/s1. Table S1. pH and colorimetric values (L*, a*, b*) of control (CTRL) and treated fresh-cut lettuce during cold storage at 4 °C for up to 10 days (mean (n = 3) ± standard deviation). Table S2. Mold and yeasts loads (log CFU g^−1^) of control (CTRL) and treated fresh-cut lettuce during cold storage at 4 °C for up to 10 days (mean (n = 3) ± standard deviation).
