# Supplementary material for: Extending More than One Week the Shelf Life of Fresh-Cut Lettuce Using Vinegar Enriched in Bioactive Compounds Encapsulated in α-Cyclodextrins
Source: Foods. 2024 Oct 1;13(19):3142. doi: 10.3390/foods13193142 (PMC11475928; doi:10.3390/foods13193142)
Supplement: Supplementary file 1 [file foods-13-03142-s001.zip › foods-3155826-supplementary.pdf]

Table S1. pH and colorimetric values (L*, a*, b*) of control (CTRL) and treated fresh-cut lettuce during cold storage at 4 °C for up to 10 days (mean (n = 3) ± standard deviation).

Time (Days)	Treatment *	pH	*L**	*a**	*b**
	CTRL	6.2±0.1^{ACa}	73.0±1.6^{Aa}	-15.2±0.5^{Aa}	30.7±1.1^{ABa}
	N-VP	5.7±0.1^{Ba}	77.4±1.3^{Aa}	-12.4±1.6^{Aab}	26.3±2.8^{Aa}
0	B-VP	6.0±0.1^{Ca}	71.9±4.6^{Aab}	-13.4±1.3^{Aa}	28.6±1.3^{ABab}
	N-VW	6.3±0.1^{Aab}	71.1±1.8^{Aa}	-14.2±1.8^{Aa}	33.1±3.7^{Ba}
	B-VW	6.4±0.1^{Aa}	74.1±1.4^{Aab}	-13.9±0.3^{Aa}	31.2±0.9^{ABac}
	CTRL	6.3±0.1^{Aa}	77.2±0.8^{Ab}	-11.7±0.4^{Ab}	26.3±2.0^{ABb}
	N-VP	6.0±0.4^{Aab}	75.3±3.3^{Aa}	-12.7±0.5^{Aa}	29.4±1.2^{ACab}
1	B-VP	6.3±0.1^{Ab}	76.1±3.1^{Ab}	-13.7±2.4^{Aa}	34.0±2.1^{Cb}
	N-VW	6.4±0.1^{Aab}	74.0±1.0^{Aa}	-11.3±1.3^{Aa}	25.0±2.5^{ABb}
	B-VW	6.4±0.1^{Aa}	73.3±2.8^{Aa}	-9.2±2.8^{Aa}	22.7±3.2^{Bb}
	CTRL	6.4±0.1^{ABb}	73.7±0,2^{Aa}	-12.1±0.5^{Ab}	26.9±0.6^{ABb}
	N-VP	6.2±0.1^{Aab}	76.8±1.8^{Aa}	-11.9±1.3^{Aab}	33.6±3.0^{Bb}
3	B-VP	6.3±0.1^{ACb}	72.3±1.8^{Aab}	-12.6±2.7^{Aa}	26.8±3.0^{Aac}
	N-VW	6.6±0.1^{Ba}	74.9±5.4^{Aa}	-12.5±2.8^{Aa}	30.0±3.7^{ABab}
	B-VW	6.5±0.1^{BCa}	73.5±2.2^{Aa}	-11.9±0.9^{Aa}	26.7±0.8^{Aab}
	CTRL	6.4±0.1^{Aab}	75.3±0.6^{ACab}	-11.8±1.6^{Ab}	28.6±0.4^{Aab}
	N-VP	6.3±0.1^{Ab}	72.7±0.8^{ABab}	-12.9±0.6^{Aa}	34.1±1.2^{Bb}
7	B-VP	6.3±0.1^{Ab}	67.0±2.9^{Ba}	-9.6±1.7^{Aa}	27.5±2.6^{Aa}
	N-VW	6.3±0.1^{Ab}	79.0±3.4^{Aa}	-13.8±1.5^{Aa}	32.8±3.3^{ABab}
	B-VW	6.4±0.1^{Aa}	69.3±4.4^{BCa}	-14.7±3.3^{Aa}	34.2±0.5^{Bc}
	CTRL	6.3±0.1^{ABab}	74.4±1.3^{Aa}	-10.8±0.6^{Ab}	28.9±0.9^{Aab}
	N-VP	6.1±0.1^{Aab}	68.3±2.2^{Bb}	-8.5±2.4^{Ab}	25.4±1.0^{Ba}
10	B-VP	6.2±0.1^{Ab}	65.9±2.8^{Ba}	-9.2±0.5^{Aa}	21.6±0.5^{Cc}
	N-VW	6.5±0.2^{Bab}	75.9±1.8^{Aa}	-14.0±2.9^{Aa}	37.1±0.6^{Da}
	B-VW	6.5±0.1^{Ba}	81.5±2.2^{Cb}	-11.4±3.5^{Aa}	29.9±2.5^{Aac}

* CTRL, control (absence of encapsulated vinegar); N-VP, normal (acidic) enriched vinegar powder; B-VP, buffered enriched vinegar powder; N-VW, normal (acidic) enriched vinegar powder washing solution; B-VW, buffered enriched vinegar powder washing solution. Different uppercase letters denote significant differences ($p < 0.05$) among different treatments for the same storage time. Different lowercase letters denote significant differences ($p < 0.05$) among different storage times for the same treatment.

Table S2. Mold and yeasts loads (log CFU g^{-1}) of control (CTRL) and treated fresh-cut lettuce during cold storage at 4 °C for up to 10 days (mean (n = 3) ± standard deviation).

Time (Days)	Treatment *	Molds	Yeasts
0	CTRL	$2.0±0.1^{Aa}$	$2.0±0.1^{Aa}$
	N-VP	$2.0±0.1^{Aa}$	$2.0±0.1^{Aa}$
	B-VP	$2.0±0.1^{Aa}$	$2.4±0.7^{Aa}$
	N-VW	$2.0±0.1^{Aa}$	$2.0±0.1^{Aa}$
	B-VW	$2.0±0.1^{Aa}$	$2.0±0.1^{Aa}$
1	CTRL	$2.0±0.1^{Ab}$	$2.0±0.1^{Aab}$
	N-VP	$2.0±0.1^{Ba}$	$2.0±0.1^{Aa}$
	B-VP	$2.0±0.1^{Ba}$	$2.0±0.1^{Aa}$
	N-VW	$2.0±0.1^{Ba}$	$2.0±0.1^{Aa}$
	B-VW	$2.0±0.1^{Ba}$	$2.0±0.1^{Aa}$
3	CTRL	$2.0±0.1^{Aa}$	$2.2±0.1^{Aab}$
	N-VP	$2.0±0.1^{Aa}$	$2.0±0.1^{Ba}$
	B-VP	$2.0±0.1^{Aa}$	$3.3±0.1^{Ca}$
	N-VW	$2.0±0.1^{Aa}$	$2.0±0.1^{Ba}$
	B-VW	$2.0±0.1^{Aa}$	$2.0±0.1^{Ba}$
7	CTRL	$2.0±0.1^{Aa}$	$2.4±0.2^{ABb}$
	N-VP	$2.0±0.1^{Aa}$	$3.5±0.7^{Bb}$
	B-VP	$2.0±0.1^{Aa}$	$5.3±0.8^{Cb}$
	N-VW	$2.0±0.1^{Aa}$	$2.0±0.1^{Aa}$
	B-VW	$2.0±0.1^{Aa}$	$2.1±0.3^{ABa}$
10	CTRL	$2.0±0.1^{Aa}$	$2.4±0.3^{Aab}$
	N-VP	$2.0±0.1^{Aa}$	$4.6±0.4^{Bc}$
	B-VP	$2.0±0.1^{Aa}$	$5.8±0.1^{Cb}$
	N-VW	$2.0±0.1^{Aa}$	$2.1±0.2^{Aa}$
	B-VW	$2.0±0.1^{Aa}$	$2.1±0.3^{Aa}$

* CTRL, control (absence of encapsulated vinegar); N-VP, normal (acidic) enriched vinegar powder; B-VP, buffered enriched vinegar powder; N-VW, normal (acidic) enriched vinegar powder washing solution; B-VW, buffered enriched vinegar powder washing solution. Different uppercase letters denote significant differences ($p < 0.05$) among different treatments for the same storage time. Different lowercase letters denote significant differences ($p < 0.05$) among different storage times for the same treatment.
